# Evaluation of an *m*Health Medication Regimen Self-Management Program for African American and Hispanic Uncontrolled Hypertensives

**DOI:** 10.3390/jpm5040389

**Published:** 2015-11-17

**Authors:** Tatiana M. Davidson, John McGillicuddy, Martina Mueller, Brenda Brunner-Jackson, April Favella, Ashley Anderson, Magaly Torres, Kenneth J. Ruggiero, Frank A. Treiber

**Affiliations:** 1 College of Medicine, Medical University of South Carolina, Charleston, SC 29425, USA; E-Mails: mcgillij@musc.edu (J.M.); treiberf@musc.edu (F.A.T.); 2 College of Nursing, Medical University of South Carolina, Charleston, SC 29425, USA; E-Mails: muellerm@musc.edu (M.M.); brunnerj@musc.edu (B.B.-J.); favela@musc.edu (A.F.); anderan@musc.edu (A.A.); torresma@musc.edu (M.T.); ruggierk@musc.edu (K.J.R.)

**Keywords:** *m*Health, iterative design, essential hypertension, patient centered

## Abstract

African Americans and Hispanics have disproportionate rates of uncontrolled essential hypertension (EH) compared to Non-Hispanic Whites. Medication non-adherence (MNA) is the leading modifiable behavior to improved blood pressure (BP) control. The *Smartphone Medication Adherence Stops Hypertension* (SMASH) program was developed using a patient-centered, theory-guided, iterative design process. Electronic medication trays provided reminder signals, and Short Message Service [SMS] messaging reminded subjects to monitor BP with Bluetooth-enabled monitors. Motivational and reinforcement text messages were sent to participants based upon levels of adherence. Thirty-eight African-American (18) and Hispanic (20) uncontrolled hypertensives completed clinic-based anthropometric and resting BP evaluations prior to randomization, and again at months 1, 3 and 6. Generalized linear mixed modeling (GLMM) revealed statistically significant time-by-treatment interactions (*p* < 0.0001) indicating significant reductions in resting systolic blood pressure (SBP) and diastolic blood pressure (DBP) for the SMASH group *vs.* the standard care (SC) control group across all time points. 70.6% of SMASH subjects *vs.* 15.8% of the SC group reached BP control (< 140/90 mmH) at month 1 (*p* < 0.001). At month 6, 94.4% of the SMASH *vs.* 41.2% of the SC group exhibited controlled BP (*p* < 0.003). Our findings provide encouraging evidence that efficacious *m*Health, chronic disease, medical regimen, self-management programs can be developed following principles of patient-centered, theory-guided design.

## 1. Introduction

Essential hypertension (EH) affects one third of adults and is an independent risk factor for stroke, cardiovascular events, congestive heart failure and renal failure [1,2,3]. African-American (AA) and Hispanic adults have 30%–60% higher rates of uncontrolled EH compared to Non-Hispanic White adults [3,4,5,6,7,8,9,10]. A leading factor responsible for uncontrolled EH is medication non-adherence (MNA) [11,12]. Medication adherence (MA) is defined as the extent to which a prescribed dose, frequency and timing of a medication are followed [13]. AAs and Hispanics exhibit 37% higher MNA rates, on average, compared to Non-Hispanic Whites [14,15,16,17,18]. This disparity is a major contributor to the ethnic/racial health disparities in EH control [5,6,7,8]. Of 133 randomized controlled trials (RCTs) directed at improving MA among uncontrolled hypertensive adults, three reviews concluded that blood pressure (BP) self-monitoring, MA reminders (often phone calls), and education/counseling—individually and/or in combination—often improved patients’ MA and resulted in small, but significant, BP declines. However, only 40%–50% of subjects achieved BP control, and improvements often deteriorated following intervention cessation [19,20,21]. In light of these findings, coupled with the staggering rates of uncontrolled EH, especially among these two ethnic groups, novel methods for increasing MA and BP control are clearly warranted.

Mobile health (*m*Health), the application of wireless technology to healthcare, is a rapidly-growing field in preventive medicine and chronic disease management [22,23,24,25]. One of *m*Health’s strengths is its ability to leverage the existing mobile technology infrastructure and the ubiquity of the mobile phone. Mobile phones are used by ~94% of US adults, and 70% of AA and 71% of Hispanic adults in the U.S. own a smartphone [26]. Our preliminary work corroborated the high prevalence of standard feature and smart phone usage among AAs and Hispanics and indicated that both groups are very receptive to *m*Health for chronic disease management [27,28].

Various technology-enabled devices and systems have emerged to address MNA [29,30,31,32,33]. Indirect monitoring devices (trays, vials and phone apps) provide pill intake reminders (e.g., blinking light, buzzer, Short Message Service [SMS]). Simple SMS reminder programs have not shown significant improvements in neither verified MA nor BP control [23]. A 2014 review of 37 electronic MA device trials involved 14 chronic conditions (e.g., EH, diabetes, asthma) with median trial duration of 5.5 months [31]. Compared to control groups, the majority of programs that provided reminder signals with or without additional feedback (e.g., LED with pill number to take, time elapsed since last dose) failed to show statistically greater MA improvements (mean adherence: 79.4% *vs.* 83.5% for control groups). Programs that integrated electronic reminder device data with healthcare delivery (e.g., majority involved health care providers (HCPs) giving MA feedback and education) showed greater improvements than control groups (mean adherence: 84.8% *vs.* 68.4%). The review included nine EH trials, and average MA for electronic device groups was only 8.2% higher than the control groups. Further, the majority of the EH trials showed no significant differences between experimental and control groups in BP levels nor percent of participants achieving BP control.

Importantly, none of the above-referenced programs incorporated a user-centered, iterative design approach guided by behavioral change theory. The *Smartphone Medication Adherence Stops Hypertension* (SMASH) *m*Health program was developed using a patient and HCP-centered iterative design process involving socio-culturally preferred and low literacy-based strategies guided by the principles of Self Determination Theory (SDT) [34,35]. SDT is framed upon developing internalized motivation (*i.e.*, autonomous regulation) in which desired behaviors are linked to one’s core values, beliefs, life goals, and one’s need to feel independent in his actions rather than feeling controlled or coerced [34,36,37,38,39,40,41,42]. Sustained adherence to health behavior changes (e.g., smoking cessation, physical activity, diet) has been achieved in programs that focused upon fostering competence (akin to self-efficacy) in engaging in required protocols and autonomous regulation for sustained engagement in the program over time. Autonomous regulation-based sustained adherence was accomplished via personalized motivational and reinforcement feedback guided by participants’ values, beliefs and goals [39,40,41,42].

The SMASH system consists of a cellular connected electronic medication device that provides reminder signals and smartphone messaging reminding patients to take their BP medications using a Bluetooth-accessible BP monitor. Culturally-attuned motivational and reinforcement text messages are sent based upon MA rates. The text messages are tailored based upon one’s values, beliefs and short and long-term life goals. For example, for 100% MA adherence the previous day or over the past several days, a religious grandmother with goals of attending more church functions and spending more time with her grandchildren would receive the message: “You are doing great! Making your body stronger and healthier to attend more bible study sessions”. If partially or completely non-adherent, this participant would receive the message: “Taking medicine is good, taking it at the right time is better! Try today. Your active grandkids need you in their future!”

The several year iterative design process of the SMASH program initially included key informant interviews and focus groups with patients and healthcare providers, which guided development of the SMASH prototype. This was followed by subsequent survey administrations that were each conducted separately with Hispanics and AAs [27,28], to integrate patients’ recommendations for further refinement of the SMASH program. We then conducted two 3-month feasibility RCTs, one with Hispanic adults and one with AA adults [43,44]. Overall findings were quite promising. They demonstrated high acceptability and adherence to the protocols, 95%–100% MA, large BP reductions, and 65%–100% JNC designated BP control compared to far lower outcomes in standard care (SC) patients [43,44,45,46]. The purpose of this study was to corroborate and extend those findings in a small-scale 6-month efficacy RCT of SMASH among AA and Hispanic adults with uncontrolled EH. We hypothesized that participants in the SMASH condition would demonstrate significantly greater increased MA and decreased BP compared to participants in the treatment-as-usual condition.

## 2. Results and Discussion

### 2.1. Results

We recruited a total of 25 Hispanic and 25 African-American subjects. All 25 AAs enrolled in the trial but three were withdrawn after discovery of previously unidentified exclusion criteria (1 end stage renal disease, 1 congestive heart failure, 1 alcoholism), and four were lost to follow-up. 22 of the 25 eligible Hispanics enrolled in the trial. The three participants who refused participation cited not wishing to disclose personal background information. Later, one participant was withdrawn due to revelation of an unidentified exclusion criteria, alcoholism, and was lost to follow-up due to leaving the state. Among the remaining 38 participants (18 AAs; 20 Hispanics) with uncontrolled EH, no statistically significant differences were observed between treatment (SMASH) and standard care (SC) control groups within each ethnic group on demographic or clinical baseline characteristics presented in Table 1. Collapsing across SMASH and SC control groups, compared to Hispanics, AAs were older, had greater % of females, were more likely to be separated/divorced, and were retired/unemployed (all *p* < 0.05). Moreover, AAs had higher baseline (*p* < 0.05), 1-month (*p* < 0.05), 3-month (*p* < 0.01) and 6-month (*p* < 0.05) SBP levels compared to Hispanics. AAs also demonstrated higher 3-month DBP (*p* < 0.05) compared to Hispanics. 

**Table 1:** Descriptive characteristics of subjects.

Variable		SMASH			SC		
		All (n = 18) ^a^	African Americans (n = 8)	Hispanics (n = 10)	All (n = 20) ^a^	African Americans (n = 10)	Hispanics (n = 10)
Age in years (Mean ± Std.)		47.50 ± 11.80	55.63 ± 10.17	41.00 ± 8.78	48.45 ± 11.32	52.9 ± 11.02	44.00 ± 10.25
Gender	Male	7 (38.9%)	1 (12.5%)	6 (60.0%)	8 (40.0%)	2 (20.0%)	6 (60.0%)
	Female	11 (61.1%)	7 (87.5%)	4 (40.0%)	12 (60.0%)	8 (80.0%)	4 (40.0%)
	Single	3 (16.7%)	0 (0.0%)	3 (30.0%)	10 (50.0%)	6 (60.0%)	4 (40.0%)
Marital Status	Married/With significant other	10 (55.6%)	3 (37.5%)	7 (70.0%)	6 (30.0%)	2 (20.0%)	4 (40.0%)
	Separated/Divorced	4 (22.2%)	4 (50.5%)	0 (0.0%)	3 (15.0%)	2 (20.0%)	1 (10.0%)
	Widowed	1 (5.6%)	1 (12.5%)	0 (0.0%)	1 (5.0%)	0 (0.00%)	1 (10.0%)
Education	High School or less	10 (55.6%)	3 (37.5%)	7 (70.0%)	12 (60.0%)	5 (50.0%)	7 (70.0%)
	Partial/college graduate	8 (44.4%)	5 (62.5%)	3 (30.0%)	8 (40.0%)	5 (50.0%)	3 (30.0%)
	$0-25K	7 (38.9%)	3 (37.5%)	4 (40.0%)	8 (40.0%)	5 (50.0%)	3 (30.0%)
Income	$25-50K	6 (33.3%)	2 (25.5%)	4 (40.0%)	5 (25.0%)	3 (30.0%)	2 (20.0%)
	$ >50K	0 (0.0%)	0 (0.0%)	0 (0.0%)	1 (5.0%)	0 (0.0%)	1 (10.0%)
	Not Reported	5 (27.8%)	3 (37.5%)	2 (20.0%)	6 (30.0%)	2 (20.0%)	4 (40.0%)
Employment	Full-/Part-time	11 (61.1%)	3 (37.5%)	8 (80.0%)	14 (70.0%)	5 (50.0%)	9 (90.0%)
	Retired/Disabled	2 (11.1%)	2 (25.5%)	0 (0.0%)	3 (15.0%)	3 (30.0%)	0 (0.0%)
	Unemployed	5 (27.8%)	3 (37.5%)	2 (20.0%)	3 (15.0%)	2 (20.0%)	(10.0%)

^a^ Represents those who completed the trial out of the final sample verified as eligible (SMASH :18/22 ;SC: 20/21).

#### 2.1.1. Changes in Clinic Systolic Blood Pressure (SBP)

**Figure 1:**
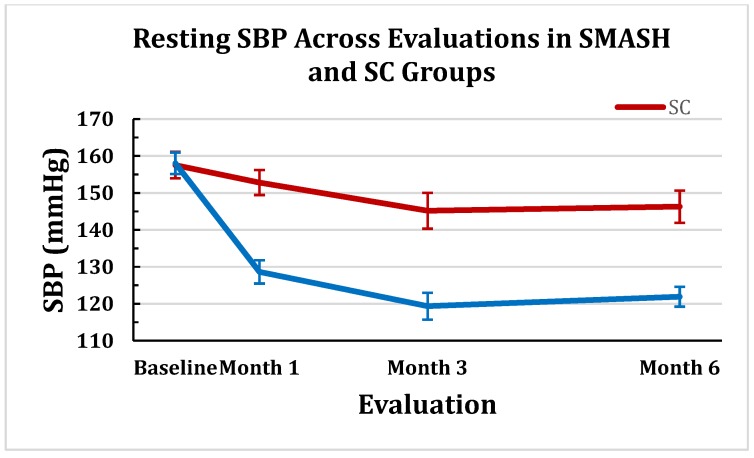
Resting SBP (± SEM) Across Evaluations in SMASH and SC Groups.

GLMM modeling of treatment over time (baseline, 1, 3 and 6 months) revealed a statistically significant time-by-treatment interaction (*p* < 0.0001). The SMASH and SC control groups were not statistically different at baseline (*p* < 0.91). The SMASH group was significantly lower than the SC group at each subsequent evaluation (all *p* < 0 .0001). Figure 1 portrays the SBP means (±SEM) between the SMASH and the SC control groups at months 1, 3 and 6. 

#### 2.1.2. Changes in Clinic Diastolic Blood Pressure (DBP)

**Figure 2:**
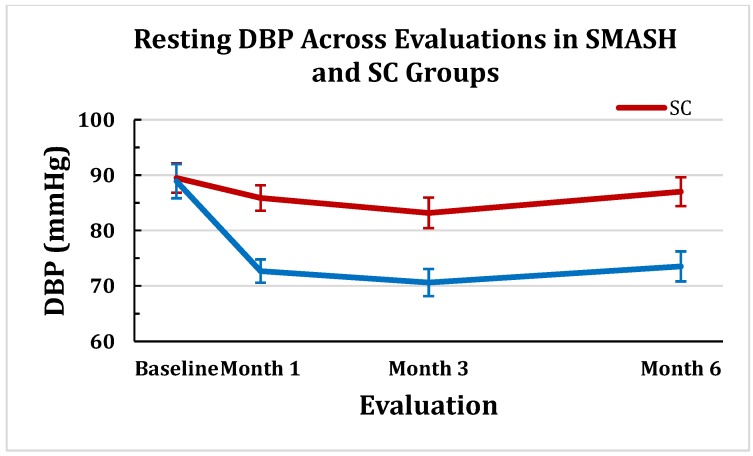
Resting DBP (± SEM) Across Evaluations in SMASH and SC Groups.

A significant time-by-treatment interaction effect was, again, observed for DBP (*p* < 0.001). Similar to SBP, the two groups were not statistically different at baseline (*p* < 0.89). The SMASH group was significantly lower than the SC group at each subsequent evaluation (all *p* < 0.001). The pattern of changes between the two groups’ DBP means (±SEM) across the 6-month trial are displayed in Figure 2.

#### 2.1.3. Changes in SBP Control

As illustrated in Table 2, no patient met JNC8 SBP control (<140 mmHg) at pre-intervention. The SMASH group exhibited significantly greater percentages of SBP control at months 1, 3 and 6. 

**Table 2:** Proportion of participants by Group with SBP controlled.

Controlled SBP	SMASH	Control	*p*-value
Baseline	00.0%	00.0%	1.000
Month 1	70.6%	15.8%	0.001
Month 3	94.4%	55.0%	0.009
Month 6	94.4%	41.2%	0.003
Sustained from month 1 through month 6	70.6%	6.3%	0.0004

#### 2.1.4. Changes in DBP Control

As illustrated in Table 3, approximately half of participants in each arm met JNC8 DBP control (<90 mmHg) at pre-intervention. The SMASH group exhibited significantly greater percentages of DBP control at months 1, 3, and 6. 

**Table 3:** Proportion of participants by Group with DBP controlled.

Controlled DBP	SMASH	Control	*p*-value
Baseline	50.0%	55.0%	1.0
Month 1	100.0%	68.4%	0.02
Month 3	100.0%	65.0%	0.009
Month 6	94.4%	76.5%	0.04
Sustained from month 1 through month 6	94.1%	37.5%	0.0008

#### 2.1.5. Feasibility Measures (Recruitment and Retention Rates, MA, BP Adherence)

We observed an overall recruitment rate of 94% (47/50) for individuals found to initially meet all inclusion criteria. All 25 AAs found to be apparently eligible were enrolled in the trial, as were 88% (22/25) of the Hispanics. Early in the trial, three AAs were removed due to undiagnosed exclusion criteria (1 active alcoholism, 1 end stage renal failure, 1 congestive heart failure). One Hispanic was also removed early in the trial due to active alcoholism.

The overall retention rate in the 6-month trial, excluding the initially-enrolled patients removed due to undiagnosed exclusion criteria, was 88.4% (38/43). It should be noted that the SMASH participants demonstrated high MA across the trial. Using the modified Russell, *et al*. [47] algorithm, MA was 92 ± 0.09 for all participants in the SMASH group and .98 ± 0.03 for AA, and 0.86 ± 0.1 for Hispanics. SMASH participants also demonstrated high protocol acceptability with overall BP self-monitoring adherence for the total expected readings being 0.98 ± 0.23. AAs averaged 0.96 ± 0.19 and Hispanics exhibited 1.00 ± 0.24. With regard to on-time BP adherence values, the SMASH group measured their BP every 3 days 86.2% ± 6% of the time. AAs averaged 9.4 ± 6.7 intervals > 3 days during the 183.4 ± 4 days they participated, resulting in on-time BP adherence of 86.6%. Hispanics averaged 8.8 ± 5.7 intervals > 3 days during the 184.1 ± 2 days they participated, yielding on-time BP adherence of 85.7%. 

#### 2.1.6. Cost of SMASH Implementation

Costs associated with implementing the SMASH program were driven primarily by equipment costs and project staff effort. The costs for using study hardware (Maya Medication Med Minder and Bluetooth-enabled BP monitor, (Maya, MedMinder Inc., Newton, MA, USA), the software application, (which enabled immediate audio and visual feedback of BP levels and automated delivery of tailored SMS motivational and reinforcement messages based upon adherence levels), was $65 per month. Participants who did not have a smart phone were loaned a smartphone with an active data package (~$63 per month). 

Project staff included two clinical research assistants, who collectively spent an average of 10 min per month per participant (~$3.7 per month) across the trial (5–8 subjects per month) responding to various patient contacts (either through telephone or SMS) and ensuring that the SMASH system operated properly. The majority of time (~30 min per subject) was spent at the outset of the trial orienting the participants to the system, helping to ensure the medication delivery schedule was programmed into the Maya portal correctly, and ensuring that the participant was able to independently and correctly operate all of the devices. There were three participants whose average session BP exceeded threshold ranges one time each during the 6 month trial. Additional time was required for the research staff to contact the participant, re-verify the out-of-threshold average BP reading level, and, subsequently, send the SMS or e-mail alert to the designated member of the healthcare team. This took 12–15 min per incident. The collective average total time that the staff dedicated to this project was ~12.5 min per month ($4.63 per month) across an average of 5–8 participants/month. Finally, healthcare providers spent at most 5 min per month per patient reviewing the weekly provider-tailored summary patient reports.

### 2.2. Discussion

The transformation of the nation’s healthcare reimbursement system to one of pay for performance (e.g., meeting best practice guidelines for risk factor levels, prevention of re-hospitalizations within 30 days of discharge) has resulted in a plethora of home-based technology enabled monitoring and delivery systems. Empirical evaluations of technology-enabled programs for chronic disease management have resulted in mixed findings with many programs failing to achieve desired improvements in risk factor levels (e.g., BP control for EH patients) [19,20,21]. A major limitation of these programs was the lack of feedback (*i.e.*, perspectives, recommendations) from major stakeholders, such as healthcare providers and/or patients, in the design phase. Further, there was often lack of utilization of behavioral change theories to foster the likelihood of sustained adherence by personalizing and tailoring the programs to the individual patients’ preferences and needs. Recent *m*Health-based chronic disease monitoring programs, which have utilized such approaches, have observed promising results [39,40,41,42]. The *Smartphone Medication Adherence Stops Hypertension* (SMASH) program is one example in which initial feasibility trials have yielded substantial improvements in MA and BP control among MNA uncontrolled hypertensive AAs and Hispanics [43,44,45,46]. 

The purpose of the present study was to corroborate and extend the early SMASH findings with a longer 6-month, small-scale efficacy RCT. We corroborated the high acceptability and usability of the SMASH program. A 95% participation rate from those confirmed as eligible is slightly higher than the average 91% participation rate observed in the two initial studies [43,44,45,46]. The retention rate for the 6-month trial was 88% compared to 100% for the 3-month feasibility trials. All drop-outs were in the SMASH group. Three of the five participants reported loss of interest in the study and 2 participants moved out of the area. The BP self-monitoring adherence rate across the 6 months was 85%, consistent with the 89% BP monitoring rates observed in the 3-month trials [44,45,46]. The SMASH group’s electronic monitoring-derived MA of 94% was consistent with the average of 95% across the earlier feasibility trials. Collectively, the time stamped MA rates across the three SMASH trials are considerably higher than those from other RCTs, which were often based upon patient self-report and/or medication possession ratios [19,20,21].

The degree of success in patients establishing JNC8 designated BP control was a primary outcome variable. The SMASH group exhibited a statistically significantly greater percentage at all three time-points with 72% of participants maintaining SBP control across the trial *vs.* 15% for the SC group. Previous BP self-monitoring and electronic-enabled MA monitoring RCTS have observed reductions in BP levels, but, on average, less than 50% of participants reached BP control [30]. As would be expected from the BP control findings, the SMASH group showed much larger reductions in both SBP and DBP compared to the SC group at each time point compared to the SC group. Across the three evaluations, the SMASH group exhibited average SBP/DBP reductions of −34.8/−12 *vs.* −9.7/−4.5 mmHg by the SC group. The SMASH group’s average SBP change at 3 months of −29.6 mmHg was much larger compared with the average -8 mmHg reduction across the collective total of 103 BP control RCTs [19,20,21].

The consistent degree of sustained JNC8-designated BP control and levels of BP reductions observed in the three SMASH program trials are remarkable given the relative simplicity of the *m*Health program compared to the multimodal face-to-face educational and cognitive behavioral skills based approaches used in previous RCTs [19,20,21]. The iterative design approach capitalized upon following preferences and suggestions of healthcare providers and AAs and Hispanics with histories of MNA. The use of self-determination theory constructs in development of the program likely fostered patient competence in being able to engage in the protocol, which they assisted in making as simple as possible to follow without significant reliance upon long-term memory. The use of tailored social reinforcement and motivational messages guided by patients’ underlying values, beliefs and life goals, also seems to have likely increased the degree of autonomous regulation, which helped increase sustained engagement in the SMASH regimen. Support for these changes comes, in part, from a recent 12 month follow-up of AA and White kidney transplant recipients who previously participated in a 3-month SMASH feasibility trial [48]. Using electronic medical records, the former SMASH patients exhibited continued significantly lower SBP levels and greater BP control compared to the former SC group (50% *vs.* 11%) at a regular clinic visit evaluation 12 months later. Additional anecdotal support comes from the current study. After the third monthly evaluation, patients were given the option to discontinue use of the medication tray and the associated intake reminder alerts if they had shown 100% adherence for at least the previous 30 days and their BP was within JNC8 guidelines. All but one patient opted to do so, and 94.4% of participants maintained BP control at the final 6-month evaluation. 

One issue frequently overlooked in *m*Health programs is the cost of implementation. The average cost per participant to engage in the SMASH program was $65 per month for those with their own smartphones and data packages. The cost for those without a smartphone was ~$128 per month. Researchers monitored emergency department (ED) utilization for the 6 months preceding and the 6 months during the trial. Only one Hispanic participant used the ED during this timeframe. The SMASH group showed a 57.5% reduction in ED use *vs.* a 5.7% reduction in the SC group compared to the 6 months prior to the trial. The average cost for an ED visit involving uncontrolled hypertension was $5,923. This estimate refers to average cost in the ED that served the overwhelming majority of visits among participants in this study. This represented an overall healthcare cost savings of $23,692 in the SMASH group *vs.* $5,923 in the SC group. The maximum cost of the SMASH program (inclusion of smartphone with data package) was ~$768 for 6 months. Assuming all participants used borrowed smartphones, the cost savings were $17,548 over the six month period. Furthermore, changes in healthcare coverage continue to be advanced with respect to coverage of remote monitoring and care delivery. As of January 2015, the Centers for Medicare and Medicaid Services (CMS) enabled bundling of two CPT codes for coverage of remote healthcare monitoring and delivery of care among Medicaid patients and Medicare patients with two or more chronic diseases. The coverage provided more than the cost per month for implementation of the SMASH program for those who have smartphones with a data plan.

Technology-based programs have the potential to facilitate treatment access and patient engagement in medical regimens as they can be tailored to the needs of the user (e.g., integration of cultural values, offered in preferred language of user), can be accessed in the home, and can overcome various economic barriers such as transportation, scheduling, and the cost of services. This is of particular importance for racial/ethnic minority groups, which have been consistently found in the literature to underutilize formal health services [49]. Over the last several years, there has been an increased awareness of the importance of cultural issues as it relates to efficacious health intervention for racial/ethnic minorities. Many experts have emphasized the importance of evidence-based treatments accounting for a patient’s cultural contexts and values, including the integration of cultural and religious values [50] as a means of increasing treatment relevance and engagement to better address the specific needs of minority cultural groups. Similarly, our previous work has found that cultural heritage, beliefs of treatment efficacy, and buy-in play a significant role in the degree to which an intervention is adopted [51,52]. To this end, the SMASH program incorporated culturally-sensitive, personalized motivational and reinforcement messages (e.g., “You are making your body strong, healthy, and ready to enjoy God’s future blessings”) based upon medication adherence rates and BP levels to motivate the participant to comply with the medical regimen. Future studies aiming at integrating culture as a means of increasing treatment engagement should consider the use of qualitative methods (e.g., individual interviews) following post-evaluation to determine which cultural considerations were considered most salient and helpful in facilitating medical regimen adherence. 

Our findings supported high acceptability and usability of the SMASH program. These results could be attributed to the multipronged approach of the SMASH program, consisting of patient-centered, *m*Health resources coupled with live interactions with providers and study staff. Chronic care models have specified that the management of disease is achieved through the collaborative efforts of providers and patients to promote self-monitoring, symptom tracking, and sharing information about health status and treatment [53,54,55,56]. Indeed, researchers have argued that along with self-monitoring, provider feedback and communication is an essential feature for *m*Health-based chronic care management, as they can provide patients with motivation and encouragement [57]. Future studies should examine the individual contributions of intervention components (*i.e.*, technology resources, provider follow-up, study-staff interactions) on patient behavior and outcomes. In addition, future studies should include patient-reported outcome measures focused on quality of life, physical and emotional well-being, and life satisfaction.

## 3. Experimental Section

### 3.1. Study Participants

Patients were eligible if they were: (1) Hispanic/Latino or African-American/Black, (2) diagnosed with and prescribed medication(s) for EH, (3) identified as having uncontrolled SBP (≥ 140 mmHg) on last visit within past 12 months with EH, (4) 21–65 years old, 5) able to measure own BP and use a smartphone; (5) negative for history of psychiatric illness, alcoholism or substance abuse, (6) negative for any other medical diagnoses, excluding type II diabetes mellitus (e.g., end stage renal disease, cancer within past two years, stroke, myocardial infarction), pregnancy or lactation or intention of becoming pregnant during the trial, (7) participating in another study or (8) able to speak, hear or understand English or Spanish. Individuals who met the eligibility criteria were contacted via telephone. Monolingual Hispanic participants were contacted by Spanish-speaking staff, and, if interested, were scheduled for a clinic BP screening. The Medical University of South Carolina Internal Review Board (IRB) approved the study protocol.

### 3.2. BP Screening and Randomization

Participants who met clinical and demographic inclusion criteria were contacted and invited to participate in the clinic BP screening. All participants completed clinic-based anthropometric and resting BP evaluations using established protocols [45] at BP screening prior to randomization, and again at months 1, 3 and 6. 

At the visit, the participant sat upright with his right arm resting on a table at heart level. An appropriately-sized cuff was applied on the right arm for a Dinamap ProCare 200 (GE HealthCare, Buckinghamshire, UK). The Dinamap has been validated using standard auscultatory methods from the British Hypertension Society and the Association for Advancement of Medical Instrumentation (AAMI) in adults [58]. The Dinamap, as well as the other BP devices used in the study, was calibrated following the manufacturer’s specifications. Readings were taken, again after a 5-minute rest, and two additional readings were taken separated by 2-minute intervals. The average of the last two reading was used in the analyses for initial eligibility and BP evaluations. EH was defined as a systolic BP ≥ 140 mmHg using Joint National Committee (JNC8) guidelines [1]. SBP was used as the selection variable since most EHs < 65 years old have systolic or combination systolic/diastolic EH and for most patients, controlling SBP also results in DBP control [59,60]. Following the pre-intervention screening evaluations and informed consent process, patients were randomized to either SC control or the SMASH intervention groups.

### 3.3. SMASH Protocol

SMASHers received a smartphone (Droid x, Motorola, Schaumburg, IL, USA), a wireless GSM electronic medication tray (Maya, MedMinder Inc., Newton, MA, USA), and a wireless Bluetooth-enabled BP monitor (A&D model UA-767PlusBT San Jose, CA, USA). The BP monitor was calibrated to satisfy the British Hypertension Society and AAMI criteria for SBP/DBP accuracy compared with standard auscultatory readings [61]. SMASHers were instructed to record their BP in the morning and evening every 3 days using the same protocol described previously.

The MedMinder medication tray, which has 28 compartments (up to four doses daily × 7 days), uses a 110 V power source, time stamps compartment use, and provides reminder signals. At the prescribed dosing time a blinking light from the specific dose compartment was activated. If that compartment had not been opened, removed, and returned for 30 minutes, a chime activated for 30 minutes. If the compartment still had not been opened, an automated reminder phone call or text message was delivered to the subject’s mobile phone. Failure to open the compartment after 90 minutes had elapsed also generated an automated email message that was delivered to the study coordinator. Subjects were sent text messages every 3 days as a reminder to measure BP in the morning and the evening using the A&D device and the resting BP protocol (described above). BP readings were automatically sent via Bluetooth to the mobile phone and from there, via cellular network, to the secure and Health Insurance Portability and Accountability Act-compliant data repository. No patient names were transmitted and no identifying information was stored on the smartphone. Via smartphone, participants received immediate audio and visual feedback on average BPs after each measurement and the application charted cumulative averages over time against threshold lines for BP control (*i.e.*, set at 140/90 mmHg). If participants were not adherent to the BP measurement schedule or if the average of the last two BP measurements were outside threshold range specified by the treating physician, the study coordinator would be alerted and would, subsequently, instruct the subject to obtain additional BP measurements. If the BP remained outside of the threshold range, the physician’s nurse manager would be alerted. We had one instance of a patient using the emergency department during the trial. 

Self-determination theory [34,35,36] was employed as the underlying behavioral change theory in this *m*Health intervention. In preliminary interviews, we found that faith, family, and friends played important roles in both African Americans’ and Hispanics’ healthcare-related behaviors. A research technician interviewed SMASHers to determine their primary interests, life values, short-term and long-term goals, and the roles of cultural values and beliefs on their healthcare behaviors. This information was used to generate personalized motivational and feedback messages that, along with other generic motivational and reinforcement messages, were delivered via the participants’ preferred mode of communication (e.g., text, email, voice mail). Message content was dictated by their MA levels from the previous day, and the messages were delivered daily for the first month and every several days thereafter. The study’s healthcare providers elected to receive twice monthly MA and BP summary reports via email. The summary reports were tailored to the specifications of the treating physicians so as to optimize their utility. 

### 3.4. Outcome Measures

As noted, SBP was a primary selection variable. The percentage of participants within group classification (SMASH *vs*. SC) who exhibited JNC8 designated SBP control (<140 mmHg) was thus used as the primary outcome variable. Percentage with DBP controlled (<90 mmHg), as well as levels of change in resting SBP and DBP were secondary outcome variables. The average of the last two BP readings from each clinic evaluation (baseline, months 1, 3 and 6) were used as dependent measures.

Feasibility measures for the SMASH trial included recruitment rates, retention rates, MA and BP adherence. MA in the SMASH cohort was calculated using a modification of the Russell *et al*. algorithm [47]. Participants were told that, in order to be considered fully compliant, all medications must be ingested within a 3-hr window centered on the prescribed dosing time. Doses taken outside that window but within a 6-hr window would receive half credit, and a dose taken outside the 6-hr window or a missed dose would receive no credit. Daily scores could range from 0 to 1, and all daily scores were averaged over the period of medication monitoring to calculate adherence. 

BP adherence was assessed using the data received from the A&D BP monitor via the smartphone. BP adherence was calculated by dividing the total number of readings received by the total number of expected readings (six readings performed every 3 days). Intervals between readings of greater than 3 days were counted and length of each recorded. On time BP adherence was determined by assessing the frequency with which participants measured their BP within 3 days after the previous reading with 100% being the maximum. The following formula was used for on-time BP adherence: 100% − (total number of time frames BP was measured at greater than a 3 day interval/total possible 3 day intervals within the study).

### 3.5. Data Analytic Plan

Primary statistical analyses were conducted for feasibility measures, including MA, BP self-monitoring adherence, and results from the health belief scale for the SMASH group using averages and standard deviations. Recruitment and retention rates were also measured. Demographics and clinical characteristics were described using means and standard deviations. Demographic and clinical baseline characteristics were compared between the intervention and control groups, as well as between ethnicity groups using chi-square tests and pooled t-tests.

To investigate the effect of the intervention on SBP and DBP over time, a generalized linear mixed model (GLMM) approach was used (PROC MIXED, SAS 9.4). GLMM was utilized to account for the correlation of measurement within participants, along with missing data. We followed the recommendations of Fitzmaurice *et al*. [62] for adjustment of baseline response levels when it is safe to assume that the two groups have the same response (SBP/DBP) at baseline for example due to randomization of participants. Models included SBP (DBP) as the dependent variable and time and a time-by-intervention interaction term as fixed effects. We further assumed that correlations between measurements within participants would decrease over time and used an autoregressive covariance structure for all models. For models without significant interaction terms, unadjusted means were reported.

Fischer exact tests were conducted to assess differences in percentages of participants by group classification who exhibited JNC8 guideline [1] defined control of SBP (<140 mmHg) at each evaluation. A similar set of analyses were conducted for control of DBP (<90 mmHg).

## 4. Conclusions

We are encouraged by the consistent pattern of the efficacy trial findings that the *m*Health medical regimen self-management program promotes and assists in maintaining medication adherence and BP monitoring, among traditionally underserved and hard-to-reach ethnic minority populations. Next steps will include a multisite efficacy/effectiveness RCT with longer post-trial follow-up evaluations to answer key questions regarding implementation, dissemination and utilization of SMASH for best practice healthcare in self-management of MNA and uncontrolled EH. 
